# Cepharanthine, a regulator of keap1-Nrf2, inhibits gastric cancer growth through oxidative stress and energy metabolism pathway

**DOI:** 10.1038/s41420-023-01752-z

**Published:** 2023-12-12

**Authors:** Yang-yang Lu, Chun-yang Zhu, Yi-xin Ding, Bing Wang, Shu-fen Zhao, Jing Lv, Shu-ming Chen, Sha-sha Wang, Yan Wang, Rui Wang, Wen-sheng Qiu, Wei-wei Qi

**Affiliations:** 1 Department of Oncology, The Affiliated Hospital of Qingdao University, Qingdao, China; 2 Biomedical Centre, Qingdao University, Qingdao, China

**Keywords:** Gastrointestinal cancer, Apoptosis

## Abstract

Cepharanthine (CEP), a bioactive compound derived from *Stephania Cephalantha Hayata*, is cytotoxic to various malignancies. However, the underlying mechanism of gastric cancer is unknown. CEP inhibited the cellular activity of gastric cancer AGS, HGC27 and MFC cell lines in this study. CEP-induced apoptosis reduced Bcl-2 expression and increased cleaved caspase 3, cleaved caspase 9, Bax, and Bad expression. CEP caused a G2 cell cycle arrest and reduced cyclin D1 and cyclin-dependent kinases 2 (CDK2) expression. Meanwhile, it increased oxidative stress, decreased mitochondrial membrane potential, and enhanced reactive oxygen species (ROS) accumulation in gastric cancer cell lines. Mechanistically, CEP inhibited Kelch-like ECH-associated protein (Keap1) expression while activating NF-E2 related factor 2 (Nrf2) nuclear translocations, increasing transcription of Nrf2 target genes quinone oxidoreductase 1 (NQO1), heme oxygenase 1 (HMOX1), and glutamate-cysteine ligase modifier subunit (GCLM). Furthermore, a combined analysis of targeted energy metabolism and RNA sequencing revealed that CEP could alter the levels of metabolic substances such as D (+) - Glucose, D-Fructose 6-phosphate, citric acid, succinic acid, and pyruvic acid, thereby altering energy metabolism in AGS cells. In addition, CEP significantly inhibited tumor growth in MFC BALB/c nude mice in vivo, consistent with the in vitro findings. Overall, CEP can induce oxidative stress by regulating Nrf2/Keap1 and alter energy metabolism, resulting in anti-gastric cancer effects. Our findings suggest a potential application of CEP in gastric cancer treatment.

## Introduction

Gastric cancer, a common cancer worldwide, is a major public health issue [1]. The overall treatment of gastric cancer, which includes surgery, radiation, targeted therapy, and immunotherapy, has significantly improved the prognosis of gastric cancer patients. However, early gastric cancer patients usually lack the telltale signs, which delays diagnosis and reduces the 5-year overall survival rate to approximately 40%. Patients with the same stage of gastric cancer may have varying treatment outcomes and prognoses due to the disease heterogeneity. Therefore, multiple target drugs with low toxicity and side effects are required to treat gastric cancer [2, 3].

Many diseases, including cancer, inflammation, diabetes, and atherosclerosis, are exacerbated by mitochondrial-induced oxidative stress. Reactive oxygen species (ROS) are produced abundantly, while antioxidant-reducing molecules such as glutathione (GSH), the antioxidant enzyme superoxide dismutase (SOD), and thioredoxin (TDX) are deficient [4]. It is worth noting that NF-E2-related factor 2 (Nrf2) has been identified as one of the primary targets for cancer therapy because it regulates oxidative stress. Nrf2 is recognized in the cytoplasm by the Kelch-like ECH-associated protein (Keap1) and degraded through the ubiquitin-proteasome pathway. When the organism’s level of oxidative stress rises, Nrf2 translocates to the nucleus and forms protein complexes with p300/CBP, musculoaponeurotic fibrosarcoma (Maf), and antioxidant response elements (ARE). This activates its target genes, including quinone oxidoreductase 1 (NQO1), heme oxygenase 1 (HMOX1), and glutamate-cysteine ligase modifier subunit (GCLM) [5]. Excessive oxidative stress is one of the principal strategies for limiting cancer development. For example, ROS and oxidative DNA damage may have anticancer effects in gastric cancer cells because the antioxidant enzyme peroxiredoxin 2 (PRDX2) is suppressed or gastrokine 2 (GKN2) is overexpressed [6, 7]. These studies indicate that targeting oxidative stress/Keap1/Nrf2 signaling is a promising anticancer strategy.

Warburg effect demonstrated that the cancer cells alter their energy metabolism to favor glycolysis in an aerobic environment [8, 9]. The reprogramming of energy metabolism is one of the top ten characteristics of cancer [10]. Cancer cells can change their metabolic status to adapt to microenvironments that do not support growth [11]. Cells can quickly respond to oxidative stress by their metabolic reprogramming. When exposed to high amounts of ROS for a brief time, cells can produce reducing molecules like glyceraldehyde-3-phosphate dehydrogenase (GAPDH) via the actions of glucose 6-phosphate dehydrogenase and the pentose phosphate pathway to mitigate the harm caused by oxidative stress [12, 13]. Therefore, the stimulation of ROS and interference with energy metabolism is one of the hotspots in cancer research.

Cepharanthine (CEP) is an alkaloid derived primarily from *Stephania cepharantha Hayata* [14]. Preliminary research indicates that CEP has antiparasitic, anticancer, antibacterial, and immunomodulatory effects [15, 16]. Importantly, CEP can significantly reduce the ability of coronavirus disease 2019 (COVID-19) to multiply in vitro [17–19]. In the present study, we emphasize the ability of CEP to combat cancers. CEP induces apoptosis in human leukemia cell lines and inhibits the growth of human hepatocellular carcinoma by activating the caspase pathway [20–22]. CEP inhibits the growth of ovarian cancer cells and cholangiocarcinomas by inhibiting the nuclear factor kappa-B (NF-kB) pathway [23]. Furthermore, CEP restored the multidrug resistance of paclitaxel-resistant A2780 cells via the PI3K/Akt signal pathway [24]. Here, our attention is on assessing CEP’s therapeutic impact on gastric cancer. CEP can limit the spread of gastric cancer cell lines, including SH101 and MKN45, according to an earlier study. CEP can control the resistance of gastrointestinal tumors to doxorubicin and work synergistically to limit the growth of gastric cancer when combined with anticancer drugs like paclitaxel. The mechanisms shown by these investigations are not obvious, though, and need more research [25–27]. CEP has the potential to be an effective treatment for gastric cancer.

## Results

### CEP exhibits cytotoxicity to gastric cancer cells

**Fig. 1:**
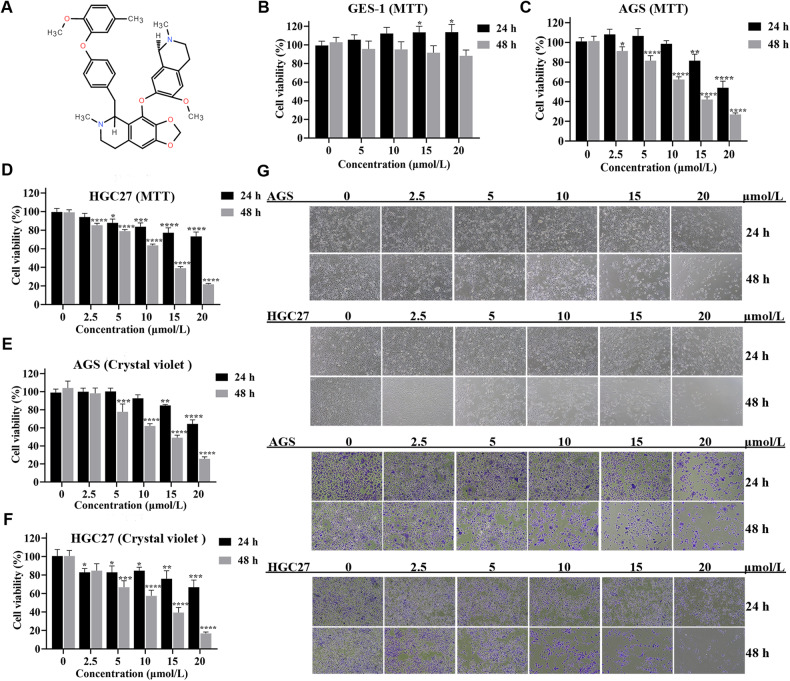
CEP is toxic to gastric cancer cells. **A** The structure of CEP. **B** Human gastric mucosal cell line GES-1 was treated with different concentrations of CEP for 24 or 48 h. The cell viability was detected by MTT assay (*n* = 4). **C**, **D** Human gastric cancer cell lines AGS and HGC27 were treated with the indicated concentration of CEP for 24 or 48 h. MTT assay (*n* = 4) was used to determine cell viability. **E**, **F** AGS and HGC27 cell lines were treated with the indicated concentration of CEP for 24 or 48 h. The cell viability was detected by crystal violet assay (*n* = 3). **G** AGS and HGC27 cell lines were treated with CEP for 24 or 48 h. Morphology was observed by live cell photography and crystal violet staining—scale bar: 100 μm. Data were expressed as mean ± SD, *p < 0.05, **p < 0.01, ***p < 0.001, and ****p < 0.0001, with significant differences from the control group.

Figure 1A depicts the structure of CEP. The MTT assay was used to assess the effect of CEP on the viability of the GES-1 cell. The results were evaluated 24 or 48 h after treatment with 5, 10, 15, and 20 μmol/L CEP. The viability of the GES-1 cell did not change significantly after 24 or 48 h of CEP treatment. CEP has low toxicity and can be tolerated by normal gastric epithelial cells at concentrations ranging from 0 to 20 μmol/L (Fig. 1B).

The MTT assay and crystal violet staining were used to analyze the effect of CEP on the viability of AGS and HGC27 human gastric cancer cells. The MTT experiment revealed that the viability of AGS and HGC27 cells decreased after CEP treatment for 24 h. The viability of cells after 48 h of treatment was 91.3% and 85.4% with 2.5 μmol/L CEP, 81.5% and 78.9% with 5 μmol/L CEP, 62.4% and 63.6% with 10 μmol/L CEP, 42.1% and 39.1% with 15 μmol/L, and 27.0% and 21.8% with 20 μmol/L CEP, respectively. Our findings demonstrated that in contrast to the control group, the viability of AGS and HGC27 cells decreased steadily as CEP concentration increased and action duration increased (Fig. 1C, D). Similar outcomes were obtained with crystal violet staining (Fig. 1E, F). In addition, we also demonstrated that CEP treatment can significantly inhibit MFC cell viability after 48 h, we used the MTT test to measure MFC cell viability. The results showed that, in comparison to the control group, the cell viability in the 5 μmol/L, 10 μmol/L, and 15 μmol/L CEP groups was 54.7%, 30.8%, and 10.8% respectively (Supplementary Fig. S1B).

The morphologies of AGS and HGC27 gastric cancer cells were also examined under a microscope while they were still alive after receiving CEP treatment and stained with crystal violet. The number of surviving cells decreased gradually as CEP concentration and drug treatment duration increased, indicating a time-dependent relationship between time and concentration. Cells developed an irregular, spherical, and bright morphology. They are consistent with the measured cell survival results (Fig. 1G). In addition, MFC cells treated with CEP for 48 h were observed by microscope imaging with similar results (Supplementary Fig. S1A).

### CEP inhibits the proliferation and migration ability of gastric cancer cells

**Fig. 2:**
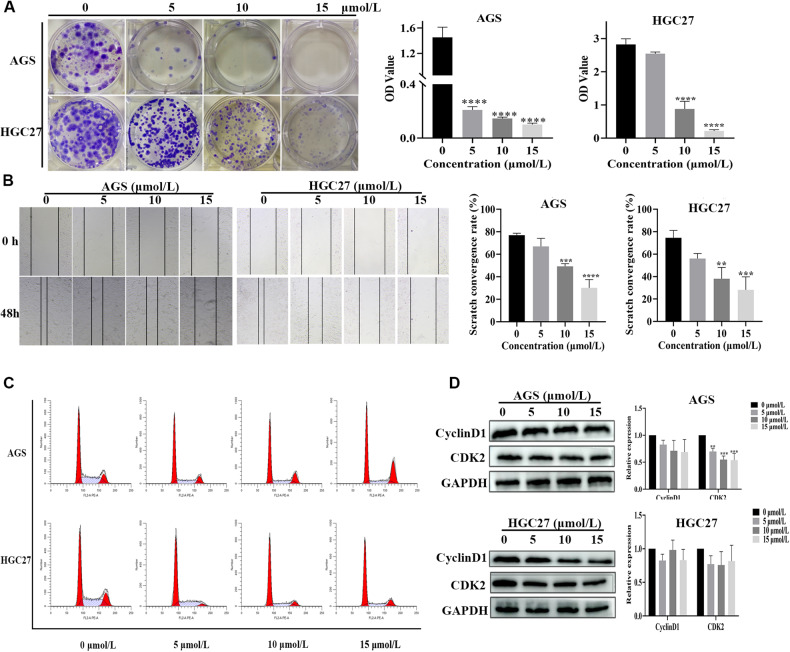
CEP suppresses gastric cancer cell growth and migration. **A** Cloning of AGS and HGC27 cells treated with CEP (5, 10, and 15 μmol/L). Quantify the clonal formation of AGS and HGC27 cells by measuring the absorbance of the solution obtained by dissolving crystal violet in glacial acetic acid (*n* = 3). **B** A scratch assay was performed to determine the migration of AGS and HGC27 cells treated with CEP (5, 10, and 15 μmol/L) for 48 h. Quantification of scratch images by calculating the area of cell migration (*n* = 3). **C** The cell cycle of AGS and HGC27 cells treated with CEP (5, 10, and 15 μmol/L) for 48 h was analyzed by flow cytometry. The percentage of AGS and HGC27 cells in different phases (*n* = 3). **D** After CEP treatment, a western blot was used to detect cyclinD1 and CDK2 levels in AGS and HGC27 cells. Protein levels were standardized using GAPDH levels, and data were expressed as mean ± SD, *p < 0.05, **p < 0.01, ***p < 0.001, and ****p < 0.0001, significantly different from the control group.

To further investigate the effect of CEP on AGS, HGC27 and MFC cell proliferation, we identified that CEP treatment could reduce the formation of AGS, HGC27 and MFC cell colonies. The Optical Density (OD value) of the solution obtained by dissolving cell colonies in glacial acetic acid indirectly demonstrated the ability of AGS, HGC27 and MFC cells to form colonies after CEP treatment. When the CEP treatment concentration was increased, the OD value of the obtained solution gradually decreased as compared to the control group (Fig. 2A, Supplementary Fig. S1C).

The ability of CEP to prevent the migration of AGS, HGC27 and MFC cells was evaluated using a cell scratch method. The cells in the control group began to migrate during the 48 h CEP treatment period, and the migration area almost completely covered the scratch area. However, as the CEP concentration increased, the area of cell migration after treatment gradually decreased. Finally, it was revealed in the cell scratch experiment that CEP significantly and dose-dependently reduced the migration of AGS, HGC27 and MFC cells (Fig. 2B, Supplementary Fig. S1D).

Flow cytometry is then used to detect cell cycle changes. The results revealed that when AGS, HGC27 and MFC cells were treated with CEP, the number of cells in the S phase decreased, and the number of cells entering the G1 phase increased as compared to the control group (Fig. 2C, Supplementary Fig. S2A). CEP also reduced cyclin D1 and CDK2 expression in AGS, HGC27 and MFC cells in a dose-dependent manner. Finally, these findings reveal that CEP can inhibit AGS, HGC27 and MFC cell growth (Fig. 2D, Supplementary Fig. S2B).

### CEP induces apoptosis in gastric cancer cells via the mitochondrial pathway

**Fig. 3:**
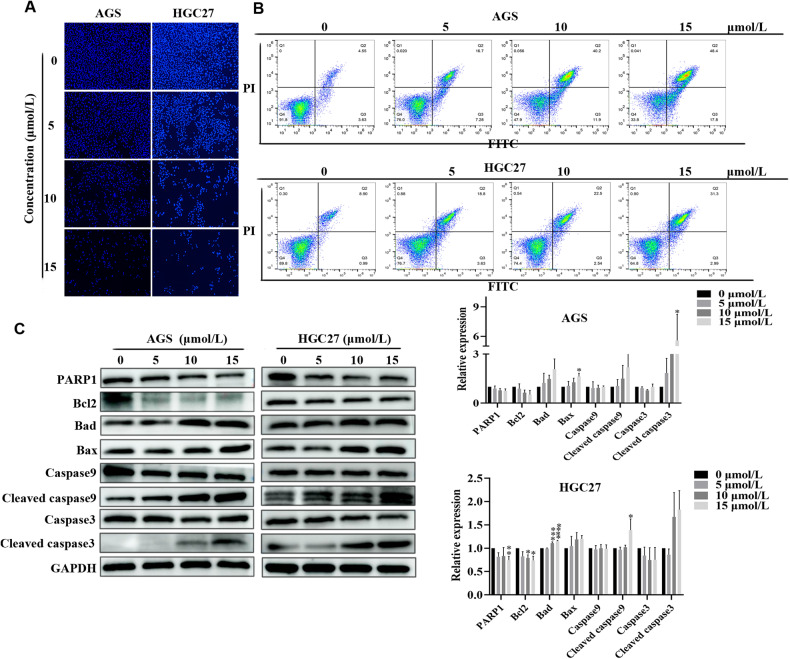
CEP induces apoptosis in gastric cancer cells. **A** Cell apoptosis detected by Hoechst 33342 staining. AGS and HGC27 cells were exposed to CEP (5, 10, and 15 μmol/L) for 48 h, and fluorescence images were taken. Scale bar: 100 µm. **B** Flow cytometry assessed cell apoptosis in AGS and HGC27 cells treated with CEP (5, 10, 15 μmol/L) for 48 h. **C** Western blot was used to determine the levels of apoptosis-related proteins in AGS and HGC27 cells after CEP treatment. CEP treatment significantly increased the ratios of Cleaved caspase-3/capase-3, Cleaved caspase-9/caspase-9, significantly increased Bax, Bad protein levels, and significantly downregulated Bcl-2 and PARP1 (*n* = 3). The protein levels were standardized using GAPDH levels, and the data were expressed as means ± SD; *p < 0.05, **p < 0.01, ***p < 0.001, and ****p < 0.0001, significantly different from the control group.

The nuclei of AGS, HGC27 and MFC cells were stained with Hoechst 33342 to determine the ultimate result of CEP therapy leading to apoptosis. The percentage of cells exhibiting granular block fluorescence increased as CEP concentration increased (Fig. 3A, Supplementary Fig. S2C). Moreover, the effect of CEP on cell apoptosis was quantified using cells stained with annexin V and PI. After 48 h of CEP treatment, there were significantly more apoptotic cells than in the control group (Fig. 3B, Supplementary Fig. S2D).

After 48 h of CEP therapy, western blot analysis was used to examine changes in the expression of proteins linked to apoptosis in AGS, HGC27 and MFC cells. It is worth noting that PARP1 and Bcl-2 protein expression were downregulated, whereas cleaved caspase-3/caspase-3, cleaved caspase-9/caspase-9, Bax, and Bad protein expression were upregulated in AGS, HGC27 and MFC cells (Fig. 3C, Supplementary Fig. S2E). These in vitro findings indicate that CEP activates the mitochondrial-dependent pathway, causing the death of gastric cancer cells.

### CEP induces the differentially expressed genes

**Fig. 4:**
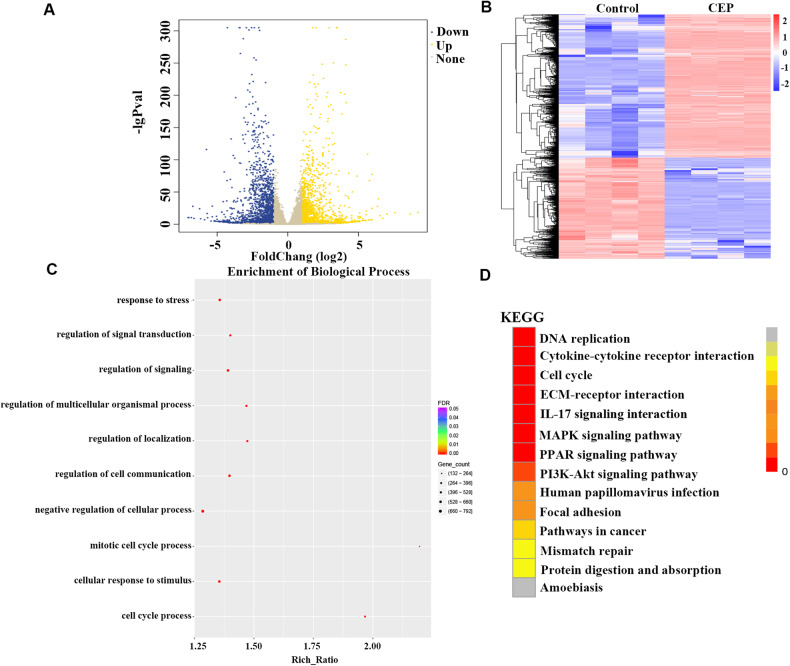
RNA sequencing results of AGS cells treated with CEP. **A** AGS cells were treated with 15 μmol/L CEP for 48 h. The RNA sequencing volcanic map revealed significant differentially expressed genes. The screening criteria for differential genes are |log2 Fold change| ≥ 1 and *q* < 0.05. Up for 1523; down for 2162. **B** The RNA sequencing heat map indicated significant differentially expressed genes. Red indicates high gene expression, while blue indicates low gene expression. The abscissa represents the sample clustering, and the ordinate represents the gene clustering. **C** GO annotation analysis of AGS cells treated with CEP compared to the control group. **D** KEGG pathway enrichment analysis of AGS cells treated with CEP revealed that different colors represented different enrichment levels, with the redder color representing more significant enrichment.

RNA sequencing was performed on AGS cells treated with 15 μmol/L CEP to investigate how CEP inhibits the growth and proliferation of gastric cancer cells, and each group was repeated four times. The global gene expression profile demonstrates that CEP regulates gene translation in AGS cells. Compared to the control group, the CEP group produced 3685 differentially expressed genes (DEGs) with 1523 upregulated and 2162 downregulated (Fig. 4A, B). The number of upregulated genes is lower than that of downregulated genes, indicating that CEP significantly inhibits gene transcription in AGS cells, resulting in cell function stagnation.

We examined these DEGs using the GO assay. This section focuses on the biological process of DEGs. GO analysis revealed that DEGs were primarily concentrated in the negative regulation of cell processes, cell communication regulation, and signal transduction regulation (Fig. 4C). We then conducted KEGG analysis. DEGs were significantly enriched in DNA replication, the cell cycle, and other pathways (Fig. 4D).

### CEP induces oxidative stress in gastric cancer cells

**Fig. 5:**
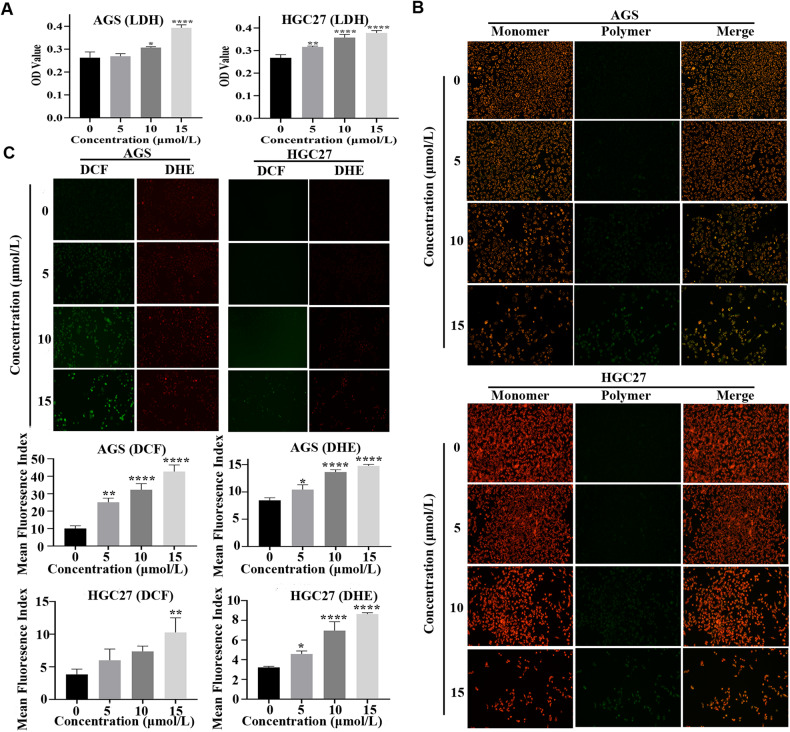
CEP increases oxidative stress levels in gastric cancer cells. **A** AGS and HGC27 were treated with CEP (5, 10, and 15 μmol/L) for 48 h. The lactate dehydrogenase (LDH) release from the cells was measured (*n* = 3). **B** The mitochondrial membrane potential was measured with JC-1 dye. AGS and HGC27 cells were treated with CEP for 48 h—scale bar: 100 μm. **C** Intracellular ROS was detected by DCFH-DA staining (green) and DHE staining (red). AGS and HGC27 cells were treated with CEP (5, 10, and 15 μmol/L) for 48 h, and fluorescence images were captured (*n* = 3). Scale bar: 100 μm. The data were expressed as means ± SD; *p < 0.05, **p < 0.01, ***p < 0.001, and ****p < 0.0001, significantly different from the control group.

Cells that have been damaged produce an excessive amount of LDH. LDH production can be measured to determine the cytotoxicity of exogenous chemicals. Add reaction reagent to cell culture supernatant to assess LDH release as per kit’s instructions. Measure the absorbance after the color of the solution has changed. The absorbance of the supernatant solution of the grown cells was 0.26, 0.27, 0.31, and 0.39 after 48 h of treatment with 0, 5, 10, and 15 μmol/L CEP in AGS cells. The absorbance for the HGC27 cell was similar, measuring 0.27, 0.32, 0.36, and 0.38, respectively (Fig. 5A). The above-mentioned findings indicate that CEP is hazardous to AGS and HGC27 cells, particularly when applied at a concentration of 15 μmol/L for 48 h.

We only examined the mitochondrial membrane potential to determine if the mitochondria are affected to determine whether oxidative stress is involved in cell CEP toxicity. The JC-1 assay was used to determine the effects of CEP on AGS, HGC27 and MFC cells. The increase in CEP concentration increased the number of JC-1 monomers (green fluorescence). In contrast, the number of JC-1 polymers (red fluorescence) decreased, indicating that mitochondrial depolarization, mitochondrial membrane potential, and mitochondrial function were all compromised (Fig. 5B, Supplementary Fig. S3B).

The amount of ROS in cells was then quantified using the DCFH-DA and DHE staining techniques. We observed an increase in the green fluorescence intensity (DCFH-DA) and red fluorescence intensity (DHE) in AGS, HGC27 and MFC cells exposed to CEP, implying that CEP increased the ROS level in these three cell lines. The data presented above demonstrated that oxidative stress was present in cells receiving CEP therapy (Fig. 5C, Supplementary Fig. S3A).

### Nrf2 is activated in oxidative stress induced by CEP

**Fig. 6:**
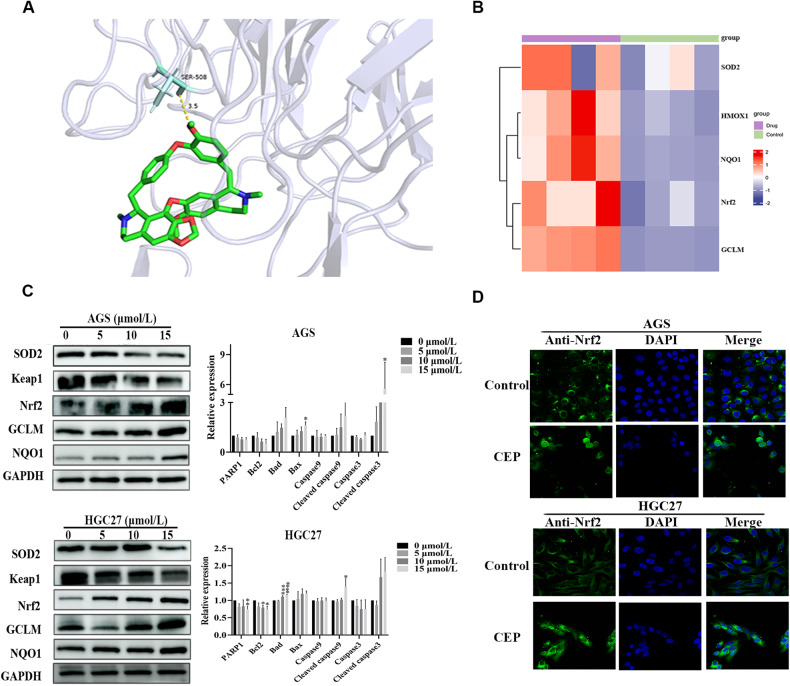
Keap1/Nrf2 is engaged in the CEP-induced cell death mechanism in gastric cancer. **A** Molecular docking of CEP with Keap1. **B** RNA sequencing revealed that the Nrf2 target genes NQO1, GCLM, and HMOX1 were upregulated after 48 h of CEP treatment of AGS cells, while the SOD2 gene was downregulated. Red indicates high gene expression, while blue indicates low gene expression. **C** AGS/HGC27 cells were treated with 5, 10, and 15 μmol/L CEP for 48 h. The protein expression of SOD2, Nrf2, Keap1, GCLM, and NQO1 was measured by western blot (*n* = 3). **D** AGS/HGC27 cells were treated with 10 μmol/L CEP for 48 h. Measurement of Nrf2 protein nuclear translocation by laser confocal microscopy. Scale bar: 50 μm. The data were expressed as means ± SD; *p < 0.05, **p < 0.01, ***p < 0.001, and ****p < 0.0001, significantly different from the control group.

**Fig. 7:**
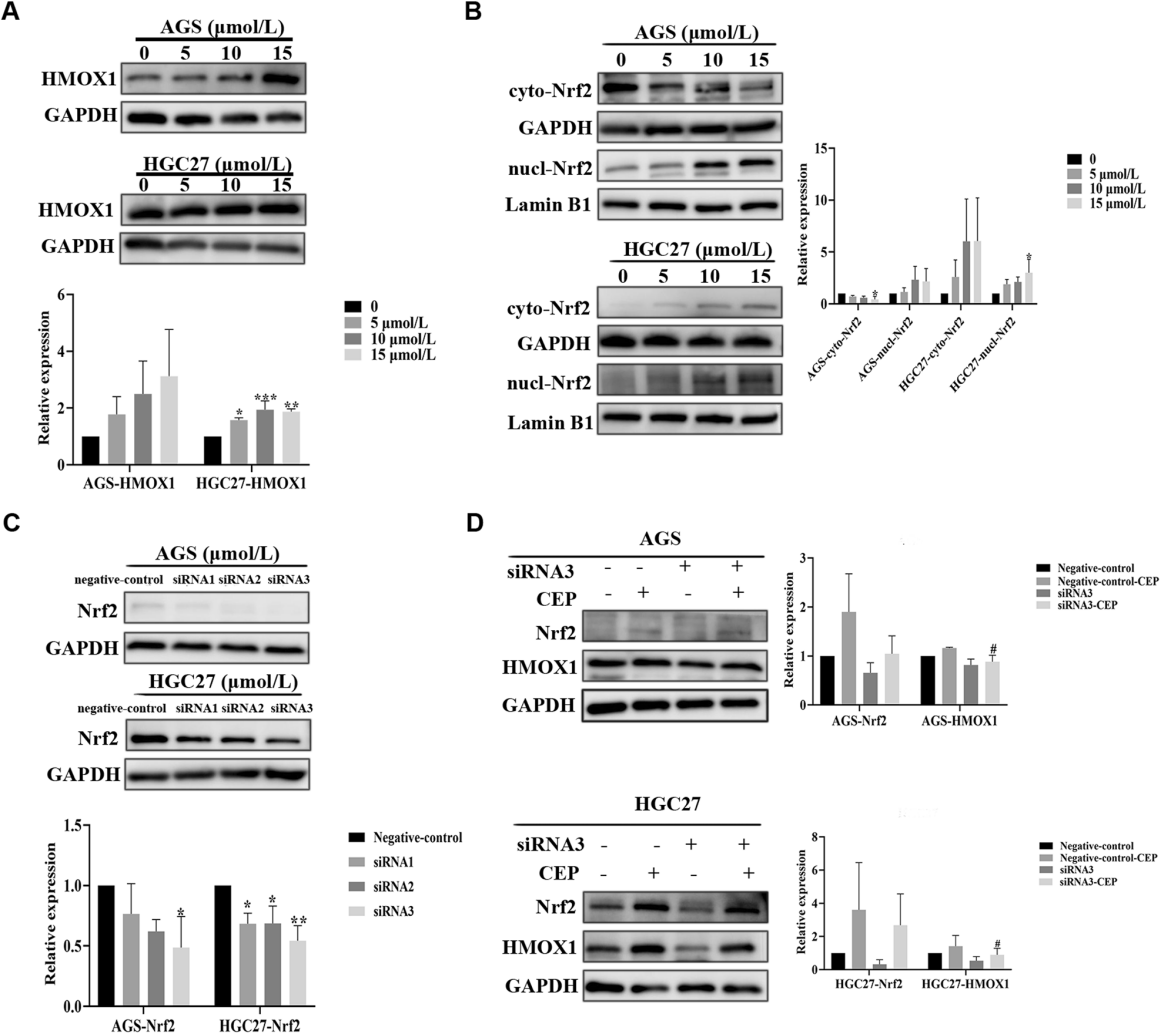
CEP can activate Nrf2 for nuclear translocation. **A** AGS/HGC27 cells were treated with 5, 10, and 15 μmol/L CEP for 48 h. The protein expression of HMOX1 was measured by western blot (*n* = 3). **B** AGS/HGC27 cells were treated with 5, 10, and 15 μmol/L CEP for 48 h. The protein expression of cyto-Nrf2 and nucl-Nrf2 was measured by western blot (*n* = 3). **C** The protein expression of Nrf2 in AGS/HGC27 cells silenced by siRNA (siRNA1, siRNA2, and siRNA3) was measured by western blot. **D** AGS/HGC27 cells were treated with 10 μmol/L CEP or siRNA3 for 48 h. The protein expression of Nrf2 and HMOX1 in AGS/HGC27 cells was measured by western blot. The data were expressed as means ± SD; *p < 0.05, **p < 0.01, ***p < 0.001, and ****p < 0.0001, significantly different from the control group. #*p* < 0.05, ##*p* < 0.01, ###*p* < 0.001, and ####*p* < 0.0001, significantly different from the Negative-control-CEP group.

We used western blot to assess SOD2 protein expression levels to understand how oxidative stress is produced. AGS and HGC27 cells had lower levels of SOD2 protein expression than control cells, indicating that oxidative stress levels increased during CEP-induced cell death (Fig. 6C). We evaluated the transcription levels of the genes Nrf2, GCLM, NQO1, and HMOX1 as well as the protein expression levels of these genes in gastric cancer cells to determine whether Nrf2 is involved in the CEP-induced oxidative stress mechanism. After 48 h of CEP treatment, Nrf2, GCLM, NQO1, and HMOX1 gene transcription or protein levels increased in AGS, HGC27 and MFC cells, but Keap1 protein levels decreased as compared to the control group (Figs. 6B, C, 7A and Supplementary Fig. S3C). Molecular docking reveals that CEP interacted with the SER-508 residues in the Keap1 protein (Score: 3.5; binding energy: −7.98) (Fig. 6A). We hypothesize that CEP affects oxidative stress and regulates the Keap1/Nrf2 axis.

We first used the immunofluorescence approach to observe the localization of Nrf2 cells following CEP treatment with the goal of further validating that CEP may activate Nrf2 and encourage the nuclear translocation of Nrf2. The outcomes showed that the fluorescence of Nrf2 protein entering the nucleus increased following CEP treatment for 48 h. Then, following CEP therapy, we discovered the expression of Nrf2 in cytoplasmic and nucleolar proteins. The findings demonstrated that Nrf2 protein expression in the nucleus gradually increased (Figs. 6D and 7B, Supplementary Figs. S3D, E).

We also performed a siRNA Nrf2 transfection experiment to strengthen the study’s credibility. All three siRNA Nrf2 target sequences could lower Nrf2 protein expression, and siRNA Nrf2-3 target sequence was chosen for further testing (Fig. 7C). HMOX1 expression decreased in the group with low Nrf2 after 10 μmol/L CEP treatment (Fig. 7D). All of these results point to CEP being able to increase Nrf2 nuclear translocation and trigger the expression of downstream GCLM, NQO1, and HMOX1 genes.

### CEP regulates energy metabolism

We hypothesize that CEP can regulate energy metabolism based on the involvement of mitochondrial damage and oxidative stress in AGS cells treated with CEP. Therefore, we try to employ the targeted energy metabolomics method for further validation. In AGS cells treated with 15 μmol/L CEP for 48 h, 68 products related to energy metabolism were identified using the LC-MS/MS platform.

**Fig. 8:**
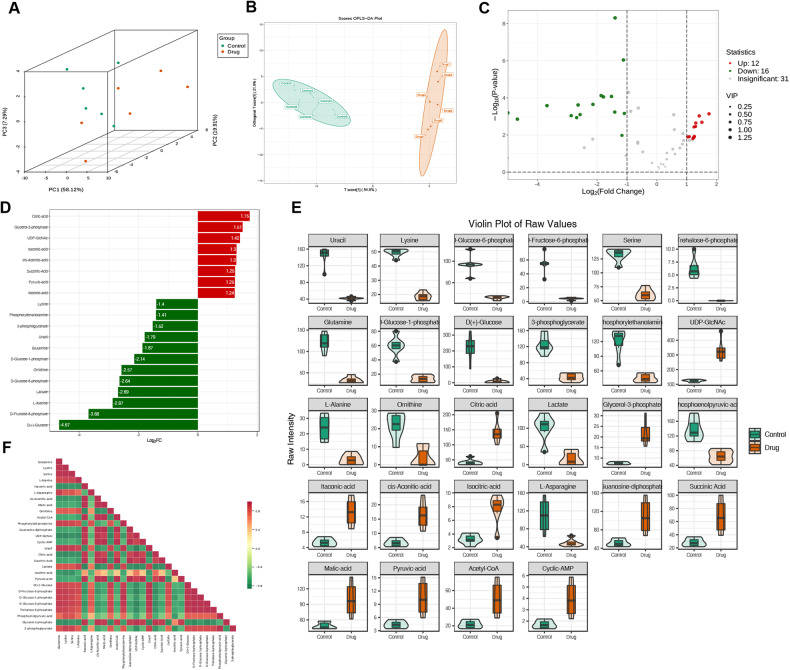
CEP can alter the energy metabolism level of AGS cells. **A** The three-dimensional principal component analysis of the untreated and drug groups (15 μmol/L CEP) can effectively observe the sample variability in each group. **B** Use OPLS-DA score maps to evaluate the effectiveness of model construction for the untreated and drug groups. **C** A volcano map depicting the differential metabolites between the untreated and drug groups. Each point in the graph represents a metabolite, with the red point representing a significantly upregulated, the green point representing a significantly downregulated, and the gray point representing a level of insignificantly changed metabolites. **D** A bar chart of differential metabolites between the untreated and drug groups. Red represents significantly upregulated, while green represents significantly downregulated metabolites. **E** A violin chart indicating differences between the untreated and drug groups. Display the data distribution and probability density. The black horizontal line in the center represents the median, while the outer shape represents the data distribution density. **F** Correlation analysis of differential metabolites in the untreated and drug groups. Red represents a strong positive correlation, while green represents a strong negative correlation.

We first performed principal component analysis (PCA) (Fig. 8A) and orthogonal partial least squares discriminant analysis (OPLS-DA) on the cell samples to be compared (Fig. 8B). The results demonstrate that the quality evaluation of each group of samples meets the requirements and can be further analyzed.

We found 12 upregulated and 16 downregulated metabolites (Fig. 8C). Figure 8D presents a bar chart of the top 20 differential metabolites. We plotted the levels of these 28 distinct metabolites (Fig. 8E). Meanwhile, the Pearson correlation analysis was used to investigate the relationship between metabolites (Fig. 8F). It is worth noting that the levels of citric acid, succinic acid, and pyruvic acid associated with the citrate cycle (TCA cycle) increased significantly. In contrast, the levels of D (+) - Glucose, D-Fructose 6-phosphate, and L-Asparagine, which are important energy sources, decreased significantly, implying that CEP can reprogram the energy metabolism in tumor cells while inhibiting tumor cell growth.

**Fig. 9:**
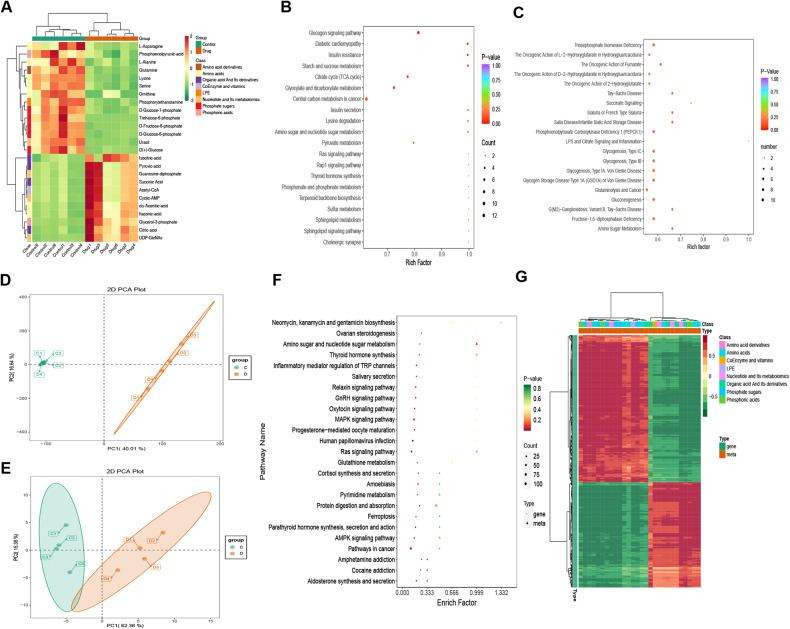
Combined analysis of RNA sequencing and energy metabolism in AGS cells treated with CEP. **A** Cluster heat map of differential metabolites between the untreated and drug groups (15 μmol/L). Red represents high content, while green represents low content. **B** KEGG enrichment analysis of differential metabolites between the untreated and drug groups. Red represents a high degree of enrichment. **C** HMDB enrichment map of differential metabolites between the untreated and drug groups. Red represents a high level of enrichment. **D** Transcriptional component analysis in the untreated and drug groups. **E** Metabolic component analysis in the untreated and drug groups. **F** The transcriptome and metabolome of the untreated and drug groups were compared, and KEGG was used to enrich the analysis. Red represents a high degree of enrichment. **G** Cluster analysis heat map of differential genes and metabolites between the untreated and drug groups. Red represents a positive correlation between genes and metabolites, while green represents a negative correlation between genes and metabolites.

We selected a KEGG metabolic pathway with at least five different metabolites and performed a cluster analysis on the content of all the different metabolites in these pathways (Fig. 9A, B). Simultaneously, we performed KEGG function enrichment and identified that these different metabolites are closely related to these pathways, such as central carbon metabolism in cancer, glucagon signaling pathway, glyoxylate, dicarboxylate metabolism, and TCA cycle. We also performed functional annotation and enrichment of the Human Metabolome Database (HMDB) and found that it is primarily enriched in triosephosphate isomerase deficiency, LPS and citrate signaling, inflammation, and fructose-1,6-diphosphatase deficiency (Fig. 9C).

### Combined analysis of RNA sequencing and energy metabolism

We selected samples with no differences for a combined analysis of RNA sequencing and energy metabolism to better understand the biological behavior of CEP-regulated gastric cancer cells. Each group was repeated four times. First, we perform PCA analyses on the RNA sequencing and energy metabolism groups (Fig. 9D, E). After determining that the sample meets the quality standards, draw the bubble map and cluster the differential genes and metabolites for correlation analysis using the KEGG path enriched by the two groups. Pathways are mainly concentrated in cancer, ras signaling pathways, MAPK signaling pathways, and ferroptosis (Fig. 9F, G).

### CEP inhibits tumor development in MFC BALB/c nude mice

To find out if CEP can stop tumor growth in vivo, we want to test our idea in AGS/HGC27 BALB/c nude mice. Unfortunately, AGS/HGC27 cell transplantation into BALB/c nude mice failed. Thus, we chose to use MFC cells to examine the anti-tumor effects of CEP in BALB/c nude mice.

**Fig. 10:**
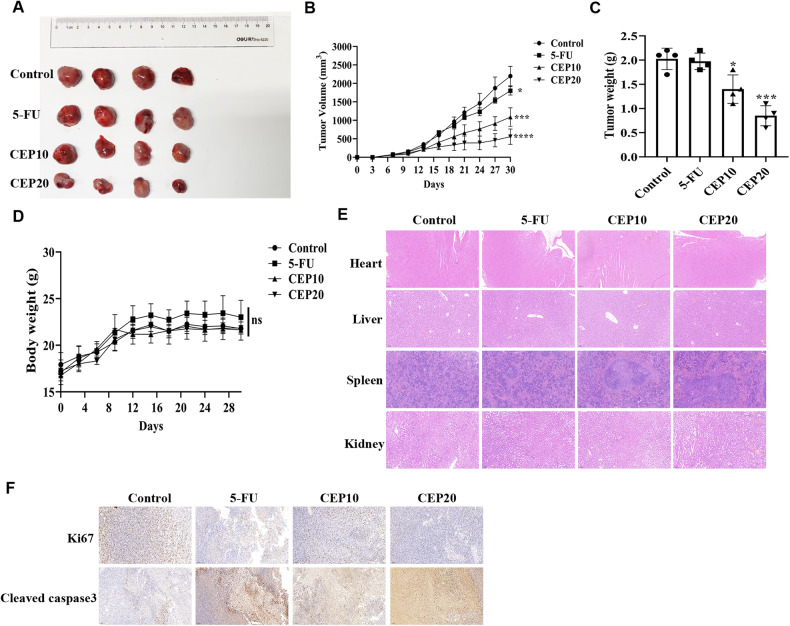
The anti-tumor efficacy of CEP was tested in vivo. **A** Images of subcutaneous tumors in MFC BALB/c nude mice, as well as tumor volume (**B**) and tumor weight (**C**) (*n* = 4). **D** The changes in body weight of MFC BALB/c nude mice treated with different drugs (*n* = 4). **E** Pathological changes (HE staining) of important organs, including the heart, liver, spleen, and kidneys in MFC BALB/c nude mice treated with different groups (*n* = 4). Scale: 100 μm. **F** Expression of Ki67 and cleaved caspase3 in tumor tissues of different groups (*n* = 3) of MFC BALB/c nude mice (immunohistochemistry). Scale: 100 μm. Data were expressed as mean ± SD, *p < 0.05, **p < 0.01, ***p < 0.001, and ****p < 0.0001, with significant differences from the control group.

We randomly divided MFC BALB/c nude mice into four groups: control, positive control (5-FU, 10 mg/kg), low-concentration treatment (10 mg/kg), and high-concentration treatment (20 mg/kg). The body weight of mice did not change significantly after 2 weeks of CEP treatment (Fig. 10D). The tumor weight and volume in the CEP and 5-FU treatment groups, however, were significantly lower than in the control group (Fig. 10A–C). We used immunohistochemistry to test different biomarkers in animal tissues to confirm the anticancer impact of CEP, including the proliferation marker Ki67 and the apoptosis marker cleaved caspase3. The findings demonstrated that tissue treated with CEP displayed a drop in Ki67 expression and an increase in cleaved caspase3, indicating a decrease in tumor proliferation ability and the incidence of apoptosis, as compared to the control group and positive control group (Fig. 10F).

In addition, we observed tumor samples isolated from mouse subcutaneous tissue with hematoxylin-eosin staining (HE staining). Compared with the control group, the pathological changes in heart, liver, spleen and kidney of mice treated with CEP were not significant, and the 5-FU treatment group also had similar results (Fig. 10E). CEP has a high safety factor and can significantly inhibit the growth of transplanted tumors in vivo.

## Discussion

Early diagnosis of gastric cancer is difficult, with high malignancy and poor prognosis. It is a common cause of death among cancer patients worldwide [28]. Chemotherapeutics such as capecitabine and 5-FU are the gold standards for treating gastric cancer. Long-term drug use puts patients at risk of developing medication resistance, organ damage, and other adverse effects. Therefore, finding therapeutic targets and potential medications to help treat gastric cancer is critical and clinically significant [29, 30].

With an increased understanding of cancer development, cancer treatment has gradually shifted from the traditional targets of cancer cell proliferation and apoptosis to the new targets of cancer cell metabolism (including energy metabolism), inflammation, oxidative stress, and immune microenvironment [31, 32]. Natural products have numerous advantages, including multiple targets, low toxicity, and side effects, which have intrigued many researchers searching for anticancer drugs [33]. Many natural products, such as paclitaxel and vincristine, have been used in clinics and benefited cancer patients [34]. Natural products have gratifying and distinct anticancer properties, whether the traditional therapeutic target or the hot new target is currently being studied. For example, rohitukine and quercetin can target CDK [35], curcumin and resveratrol can target cell apoptosis [36, 37], wedelolactone and parthenolide can target inflammation [38, 39], genistein can target glycolysis [40], and icariin and baicalin can target immunity [41, 42].

CEP is a bisbenzylisoquinoline alkaloid compound used to treat granulocytopenia, malaria, and other conditions [43–45]. The ability of CEP to significantly inhibit coronavirus disease 2019 (COVID-19) has recently been demonstrated, prompting us to reconsider its anticancer potential [17]. It has been reported that it can prevent cancers such as liver cancer [46], lung cancer [47], breast cancer [48], and ovarian cancer [24]. In the present study, we discussed the therapeutic effect of CEP on gastric cancer.

On the traditional target, we demonstrated that CEP could significantly inhibit the proliferation and migration of gastric cancer cells (AGS, HGC27, MFC) in vitro and regulate the cell cycle without damaging normal gastric epithelial cells GES-1. In vivo studies revealed that CEP significantly delayed the tumor growth of MFC cells BALB/c nude mice, comparable to the effect of the clinical first-line drugs 5-FU and HE staining of important tissues and organs of mice confirmed the safety of CEP. The pharmacological activity of CEP in vasodilation should be noted. This appears to be harmful for cancer patients’ long-term use. The assessment of CEP localization in the management of gastric cancer is challenging and necessitates our extra focus [49].

There are several types of cell death, including necrosis [50], immunogenic cell death [51], apoptosis [50], ferroptosis [52], and cuproptosis [53]. In this section, we will focus on the most common and well-studied mode of death, apoptosis. Cleaved caspase-9 and -3 are important markers of cell apoptosis. Apoptotic proteins, such as Bax and Bad, alter the permeability of the mitochondrial membrane by transporting it to the outer membrane and activating caspase-9 to form apoptotic bodies via complex regulatory mechanisms. Caspases 3 and PARP1 were then activated to promote cell apoptosis [54]. Our data, whether intuitive (cell fluorescence) or qualitative (flow cytometry), have demonstrated that CEP induces apoptotic in gastric cancer cells in vitro.

We identified that Bcl-2 and PARP1 protein expression was significantly downregulated in the CEP-inhibited tumor growth model, whereas Bax, Bad, cleaved caspase-3, and -9 protein expression was significantly upregulated. These findings indicate that CEP may have anti-gastric cancer effects through the mitochondrial apoptosis pathway Bax/Bad/Bcl2/caspase-9/caspase-3.

We are using oxidative stress as a target to highlight the internal machinations of cell apoptosis. Cell ROS increases when mitochondria are damaged and redox homeostasis is disturbed. Excessive ROS can cause apoptosis, necrosis, ferroptosis, and oxidative stress [55]. Following the discovery that chemotherapy drugs (adriamycin and cisplatin) can kill cancer cells by promoting the accumulation of ROS, natural products appear to be more inclined to increase the level of ROS to play an anti-tumor role [56].

Baicalein induces apoptosis in bladder cancer cells by increasing intracellular ROS and Bax protein expression and decreasing Bcl-2 expression [57]. Chlorogenic acid is linked to cycle arrest via ROS, which has a negative effect on human colon cancer cells [58]. Similarly, our findings revealed that CEP significantly increased the level of ROS in gastric cancer cells. We believe that increased oxidative stress is the cause of apoptosis in gastric cancer cells.

We focused on the Nrf2/Keap1 pathway as an oxidative stress mechanism. The redox status of cells is controlled by a transcription factor named Nrf2. Keap1 and Nrf2 bind to the cytoplasm during normal cell activity and this binding is recognized by cu3E3 ubiquitin ligase, degrading Nrf2 protein by ubiquitin-proteasome. Until now, under conditions of oxidative stress, Keap1 is deactivated, and the probability of Nrf2 being ubiquitinated is reduced, increasing the level of the Nrf2 protein. Moreover, once inside the nucleus, Nrf2 interacts with the small Maf protein heterodimer and the ARE to initiate the transcription of target genes (HMOX1, GCLM, and NQO1) and maintain redox homeostasis [59].

Several natural products, like curcumin and lycopene, can disrupt Keap1 and activate Nrf2 during the process of causing cancer cell death [60]. In the present study, we initially used molecular docking to explore the potential of combining CEP and Keap1. The findings demonstrated that SER-508 residue of Keap1 and CEP might interact. Western blot further revealed that CEP might deteriorate Keap1 while maintaining Nrf2 protein expression. RNA sequencing also increased the transcriptional level of Nrf2 target genes like HMOX1, GCLM, and NQO1. We also measured HMOX1, GCLM, and NQO1 protein levels to see if the results were consistent with the transcriptional level. In addition, we observed the localization of Nrf2 in cells by immunofluorescence and measured the expression level of Nrf2 in the cytoplasm and nucleus. To make the conclusion more convincing, Nrf2 siRNA experiments were conducted to observe the expression of HMOX1 in CEP-treated cells. It is reasonable to conclude that Nrf2 is activated during CEP-induced cancer cell death.

However, the mechanism by which CEP activates Nrf2 expression in gastric cancer cells is unknown. Although CEP treatment increases Nrf2 mRNA levels, whether a decrease in proteasome degradation accompanies this requires further investigation.

We attempted to investigate energy metabolism at the new target. We first performed RNA sequencing analysis to better understand the role of CEP in regulating gastric cancer cells. The findings indicated that CEP might regulate inflammation, cell metabolism, the MAPK signal pathway, the PI3K-AKT signal pathway, and other pathways. Cell metabolism is our primary concern. Tumor cells adjust their metabolism or gene expression to maintain healthy ROS levels to withstand oxidative stress. This process is inextricably linked to energy metabolism. As a fundamental aspect of life activities, energy metabolism includes glucose, glutamine, and fatty acid metabolism. Blocking the metabolism of cancer cells would be a successful cancer treatment [61].

We detected 12 upregulated and 16 downregulated energy metabolites through targeted energy metabolism detection. Citric acid, succinic acid, and pyruvate related to the TCA cycle increased significantly, while the D (+) - glucose, D-fructose-6-phosphate, and L-aspartic acid are important energy sources, decreased significantly.

Enrichment analysis revealed that CEP is intimately linked to central carbon metabolism, dicarboxylic acid metabolism, and the TCA cycle in controlling the proliferation of gastric cancer cells. This indicates that CEP can alter the way gastric cancer cells process energy. It is important to note that biological processes are distinguished by their complexity and integrity. It is challenging to fully understand the biological mechanism and biological network control of CEP regulating gastric cancer with a single omics. Data difficulties caused by missing data, noise, and other factors can be overcome when evaluating single omics data. Moreover, integrating several omics data sets for analysis facilitates the study of phenotype and biological process regulation mechanisms in biological models.

We identified that CEP was indeed involved in suppressing gastric cancer and modulating the MAPK signal pathway by the combined analysis of RNA sequencing and energy metabolism. Ferroptosis, a novel form of death groping, has recently received much attention. Ferroptosis is a type of programmed death caused by an excess of iron and an increase in active oxygen species, which causes the peroxidation of phospholipids rich in polyunsaturated fatty acids in the cell membrane. The main enzyme of the antioxidant system, GPX4, will be reduced throughout this process [62]. Our collaborative analysis revealed that CEP could regulate ferroptosis in gastric cancer cells, which warrants further study.

## Conclusion

In summary, this research reveals that CEP regulates energy metabolism to inhibit cell growth and responds to oxidative stress by regulating the Keap1-Nrf2 axis, which results in cell cycle arrest and apoptosis in gastric cancer cells. Further study supports that CEP prevents tumor formation in MFC BALB/c nude mice. This work reexamined CEP’s pharmacological efficacy in suppressing gastric cancer growth. However, because CEP’s safety in the human body has not been fully evaluated, it will take a long time and a significant amount of research to fully examine its application in the treatment of cancer patients.

## Materials and methods

### Cell culture and reagents

Human gastric cancer cell lines (AGS and HGC27) and human gastric mucosal cell line GES-1 were obtained from the Cell Bank of Type Culture Collection of the Chinese Academy of Sciences (Shanghai, China). Mouse gastric cancer cell line MFC was purchased from Procell Life Science & Technology Co., Ltd (Wuhan, China). These cell lines were identified through STR and tested negative for Mycoplasma. These cell lines were cultured in Roswell Park Memorial Institute (RPMI)-1640 medium (HyClone, USA) supplemented with 10% fetal bovine serum (FBS) (HyClone, USA), 100 U/mL penicillin (HyClone, USA) and 100 µg/mL streptomycin (HyClone, USA) at 37 °C with 5% CO_2_.

Cepharanthine (CEP, C427251), obtained from Aladdin (Shanghai, China), was dissolved in dimethyl sulfoxide (DMSO; Solarbio, China) to prepare a 90 mM stock solution and then diluted with RPMI-1640 medium before use. 5-Fluorouracil (5-FU, F408985), 3-(4,5-dimethylthiazolyl-2)-2,5-diphenyltetrazolium bromide (MTT; M158055) and Crystal violet (C110703) were purchased from Aladdin. 2′,7′-dichlorodihydrofluorescein diacetate (H2DCFDA; D6883), Dihydroethidium (DHE; 37291) were purchased from Sigma-Aldrich (USA). JC-1 Mitochondrial Membrane Potential Assay Kit (40706ES60), Annexin V-FITC/ Propidium Iodide Apoptosis Assay Kit (40302ES60), Cell Cycle and Apoptosis Analysis Kit (40301ES50), Hoechst 33342 (40732ES03), and Hieff Trans ® SiRNA/miRNA in vitro transfection reagent (40806ES) were purchased from Yeasen Biotechnology Co., Ltd (Shanghai, China). Lactate dehydrogenase (LDH) cytotoxicity test kit (C0016) and nuclear and cytoplasmic protein extraction kit (P0027) were purchased from Beyotime Biotechnology (Shanghai, China).

HRP Goat Anti Mouse IgG (H+L) (AS003), HRP Goat Anti Rabbit IgG (H+L) (AS014), antibodies against poly (ADP-ribose) (PARP1) (A19612), caspase-3/cleaved caspase-3 (A11040), caspase-9/ cleaved caspase-9 (A22636), Bad (A19595), Bax (A19684), Bcl-2 (A200777), cyclin D1 (A19038), CDK2 (A0094) and GAPDH (AC001) were purchased from ABclonal Technology Co., Ltd (Wuhan, China). and SOD2 (#13141) were purchased from Cell Signaling Technology. Nrf2 (ab62352), Keap1 (ab227828), GCLM (ab126704), NQO1 (ab80588), HMOX1 (ab189491) and Lamin B1 (ab133741) were purchased from Abcam. Goat Anti-Rabbit IgG (H+L) (Cyanine3 conjugated) (E-AB-1010) was purchased from Elabscience Biotechnology Co., Ltd (Wuhan, China). Antibody against Nrf2 (YT3189) was purchased from ImmunoWay Biotechnology Company.

### Cell viability assay

MTT assay: AGS, HGC27, MFC, and GES-1 were seeded in 24-well plates at 3.5 × 10^4^/well, 3.3 × 10^4^ /well, 5 × 10^4^/well, and 5 × 10^4^/well, respectively. All cell lines were then exposed to CEP at different concentrations for 24 or 48 h. Then, 500 µL 0.5 mg/mL MTT prepared with the culture medium without FBS was added to each well and incubated in the incubator for 2 h. Then, blue-purple crystal formazan was dissolved by adding an equal volume of DMSO. We measured absorbance at 490 nm with a full-function microplate detector (BioTek, USA) and calculated cell viability using the formula (The cell viability (%) = A490 of the treatment group/A490 of the control group).

Crystal violet assay: AGS (3.5 × 10^4^), HGC27 (3.3 × 10^4^), MFC (5 × 10^4^), and GES-1 (5 × 10^4^) were added to each well of the 24-well plate. The cell lines were incubated with varying doses of CEP for 24 or 48 h in the CO_2_ incubator (Thermo, USA). Cells were fixed in 4% paraformaldehyde for 6 h at room temperature before being stained with crystal violet solution. We used a full-function microplate detector to detect the solution’s absorbance at 570 nm after adding 500 µL glacial acetic acid to each well. Then, we applied the formula (The cell viability (%) = A570 of the treatment group/A570 of the control group) to assess cell viability.

### Colony formation assay

Each well of the 6-well plates was filled with 1000 cells, which were then treated with CEP and had the medium replaced every two days while incubating in a CO_2_ incubator (37 °C, 5% CO_2_). 14 days later, the cell colony was fixed in 4% paraformaldehyde at room temperature and dyed and photographed with crystal violet. Add 1 mL of glacial acetic acid solution to each well to completely dissolve the crystal violet. Then, use the full-function microplate detector.

### Migration assay

Inoculate the cells in a 24-well plate and maintain stable cell culture. When the cell density exceeds 90% of the area of each well, using 200 µL tips produces a wound on the cell surface. The adhering cells were grown in the medium for 4 h after the floating cells were washed with phosphate-buffered saline (PBS). After CEP was applied in various doses and incubated for 48 h, the wound width was measured using a microscope (Nikon, Japan). The data were analyzed using ImageJ software.

### Cell cycle assay

Double-stranded DNA can be coupled with Propidium Iodide, a fluorescent dye for double-stranded DNA, to identify the cell cycle. Trypsin was used to collect the cells after 48 h of CEP exposure. To prepare cell samples for future use, rinse them once with precooled PBS, centrifuge them, gently mix them with precooled 70% ethanol, and fix them overnight at 4 °C. After 30 min of dark incubation at 37 °C, the cell samples were stained with a staining solution containing Propidium Iodide and RNase A, and analyzed through flow cytometry.

### LDH release assay

Inoculate AGS and HGC27 cells into 24-well plates to maintain an 80–90% cell density throughout the test. Following stable incubation in an incubator for 24 h, CEP was added for treatment for 48 h. After removing the supernatant from each well and adding reaction chemicals in accordance with the kit’s instructions, measure the absorbance of each group at 490 nm with a full-function microplate detector.

### ROS assay

H2DCFDA (10 mmol/L) and DHE (10 mmol/L) were diluted to a final concentration of 10 µmol/L in the FBS-free RPMI-1640 medium. After CEP treatment, the cell lines were washed with PBS 1–2 times, and the prepared DCFH-DA was then added to the cell plate and incubated for 30 min with the cells. A fluorescent inverted microscope measured the ROS level.

### Mitochondrial membrane potential assay

JC-1 is an ideal fluorescent probe for detecting intracellular mitochondrial membrane potential changes. JC-1 aggregates in the mitochondrial matrix to form a polymer that emits a strong red fluorescence in normal mitochondria. Because of the reduction or loss of mitochondrial membrane potential, JC-1 can only exist as a monomer in the cytoplasm that emits green fluorescence. The JC-1 dye working solution consisted of ultrapure water and 5X JC-1 staining buffer. It was then added to the cell culture plate in equal proportion to the RPMI-1640 medium. After 30 min in a CO_2_ incubator, rinse 2–3 times with JC-1 dye buffer. The mitochondrial membrane potential was determined using a fluorescent inverted microscope.

### Hoechst 33342 analysis

Hoechst 33342 is a blue fluorescent dye that can cross cell membranes. It produces intense blue fluorescence after embedding double-stranded DNA. The Hoechst 33342 dye solution was diluted to 1 µg/mL in FBS-free 1640 medium. Remove the cell culture solution and wash it with PBS 1–2 times after 48 h of CEP treatment of cell lines. The cell lines were treated with Hoechst 33342 dye solution for 30 min before the fluorescence intensity was measured with a fluorescent inverted microscope.

### Apoptosis assay

To detect early apoptotic cells, Annexin V (35–36 kDa), a Ca^2+^-dependent phospholipid binding protein, can attach to phosphatidylserine exposed on the surface of the cell membrane. Moreover, Propidium Iodide is a dye for nucleic acids. Cell membranes of normal or early apoptotic cells cannot be penetrated. However, necrotic and late apoptotic cell membranes can be dyed red.

Several cell lines were digested with 0.25% trypsin (without EDTA) and centrifuged (4 °C, 1000 rpm, 3 min) after receiving CEP treatment for 48 h. The cells were washed twice with PBS and suspended in 1 × Binding Buffer. A total of 100 µL cell suspension should be thoroughly mixed with 5 µL Annexin V-FITC before being incubated at room temperature and away from light for 5 min. At the end of the incubation period, 10 µL Propidium Iodide staining solution and 400 µL PBS were added and immediately started flow detection.

### Immunofluorescence

AGS, HGC27, and MFC cells were inoculated on cover glass in 24-well plates at 3.5 × 10^4^/well, 3.3 × 10^4^ /well, and 5 × 10^4^/well, respectively. After 48 h of treatment with CEP, the cells were fixed for 30 min with 4% paraformaldehyde, penetrated with 0.2% TritonX-100 for 10 min, sealed with blocking solution for 1 h, and incubated with antibody against Nrf2 (ImmunoWay, 1:200) overnight. The Goat Anti-Rabbit IgG (H+L) (Cyanine3 conjugated) was then incubated, and DAPI staining was performed. Transfer images to a laser scanning confocal microscope (Leica, Germany).

### Small interfering RNA (siRNA) transfection

Suzhou Haixing Biotechnology Co., Ltd. (Jiangsu, China) synthesized the siRNA (siNrf2–1 target sequence: 5′–CAGUCUUCAUUGCUACUAAUCTTGAUUAGUAGCAAUGAAGACUGTT–3′; siNrf2–2 target sequence: 5′–GACAGAAGUUGACAAUUAUCATTUGAUAAUUGUCAACUUCUGUCTT–3′; siNrf2–3 target sequence: 5′–CAUUGAUGUUUCUGAUCUAUCTTGAUAGAUCAGAAACAUCAAUGTT–3′; negative control target sequence: 5′–UUCUCCGAACGUGUCACGUTTACGUGACACGUUCGGAGAATT–3′). Inject AGS (1.2 × 10^5^/well) and HGC27 (2 × 10^5^/well) cells into a six-well plate. After 24 h of cell culture in the incubator, use Hieff Trans ® SiRNA/miRNA in vitro transfection reagent to transfect siRNA into cells, incubate for 6–8 h, and then change the culture medium to continue cultivation for 48 h before proceeding with future operations.

### Separation of cytoplasmic and nuclear proteins

To separate cytoplasmic and nuclear proteins from AGS, HGC27, and MFC cells, use cytoplasmic protein extraction reagent A, cytoplasmic protein extraction reagent B, and nuclear protein extraction reagent. For the operation steps, follow the reagent kit’s instructions. Separately quantify the isolated proteins and then perform a western blot to determine the degree of Nrf2 expression.

### Western blot analysis

AGS, HGC27 and MFC cell lines were infused into cell culture dishes and grown for 24 h. Place the cell protein heated at 100 °C for 10 min. After half an hour of 80 V electrophoresis and 1 h of 120 V electrophoresis in one-step rapid preparation of PAGE gel kit (Shanghai, China), the protein was transferred to a polyvinylidene difluoride (PVDF) (Merck Millipore, Billerica, USA) at 300 mA for 2 h.

PVDF was sealed with 5% skimmed milk for 2 h at room temperature, then incubated overnight at 4 °C with antibodies against poly (ADP-ribose) (PARP1), caspase-3/cleaved caspase-3, caspase-9/cleaved caspase-9, Bad, Bax, Bcl-2, cyclin D1, CDK2, SOD2, GCLM, NQO1, HMOX1, Nrf2, Keap1, GAPDH, Lamin B1. The PVDF membrane is then incubated for 2 h at room temperature with either HRP Goat Anti Mouse IgG (H+L) or HRP Goat Anti Rabbit IgG (H+L). The protein signal was detected using an electrochemical luminescence (ECL) detection kit (Shanghai, China), and the signal intensity was quantified with ImageJ software.

### RNA sequencing and differential gene enrichment analysis

AGS cells were lysed with TRIzol Reagent (Seville Biotechnology, Wuhan, China) after 48 h of incubation with 15 µmol/L CEP to obtain total RNA. The RNA samples were qualified using a NanoPhotometer^®^ spectrophotometer (Thermo Fisher, USA) and an Agilent 2100 RNA Nano 6000 Assay Kit (Agilent Technologies, CA, USA). The RNA sequencing was performed by Annoroad Gene Tech. Co., Ltd. (Beijing, China). We used DESeq2 for gene differential expression analysis and plotted with the R package. The database acknowledges Gene Ontology (GO) and Kyoto Encyclopedia of Genes and Genomes (KEGG) pathways used for annotation, visualization, and integrated discovery (DAVID version 6.8.) (https://david.ncifcrf.gov/).

### Energy metabolism and combined RNA sequencing analysis

AGS cells were collected for the control and drug groups (15 µmol/L CEP) and rapidly frozen with liquid nitrogen. Six duplicate samples were selected for each group. Send the samples to Yiyan Technology Co., Ltd. (Beijing, China) for LC-MS/MS-based targeted energy metabolism detection and analysis (heat map, principal component analysis (PCA), and orthogonal partial least squares discriminant analysis (OPLS-DA)). We also paid the company to analyze the RNA sequencing data and the measured metabolites in combination.

### Animal experiments

Female BALB/c nude mice (4–6 weeks old) were purchased from Weitong Lihua Experimental Animal Technology (Beijing, China) and raised under the guidance of the Ethics Committee of The Affiliated Hospital of Qingdao University (NQ: AHQU-MAL20220819). All mice were kept in an environment with no specific pathogens, comfortable temperature, and humidity for 1 week. MFC cells (2.5 × 10^5^) were subcutaneously injected into BALB/c nude mice. When the tumor volume was measured with a vernier caliper and reached 50 mm^3^, mice were divided into four groups randomly: control, 5-FU (10 mg/Kg), CEP (10 mg/Kg), and CEP (20 mg/Kg) groups with four mice in each group. Inject solvent or drugs into the abdominal cavity of mice every 2 days and measure their weight and tumor volume (tumor volume (mm^3^) = length × width^2^ × 0.5) before each drug treatment. After 3 weeks, the mice were killed after collecting their blood, tumor tissue, heart, liver, kidney, and spleen, and photographed, weighed, fixed with paraformaldehyde, and frozen. The processed tissues were sent to Wuhan Servicebio Biotechnology Co., Ltd. (Wuhan, China) for pathological examination.

### Statistical analysis

The in vivo and in vitro tests that we conducted had randomization and blinding. Every statistic is expressed as mean ± SD. “*n*” denotes the independent sample size and is not repeated in a single experiment. One-way analysis of variance (ANOVA) test was used to examine differences between groups using GraphPad Prism 8.0. *P* < 0.05 is regarded as significant.

### Supplementary information

Supplementary Material

## Data Availability

The article and supplementary materials contain the data for this investigation. The corresponding author can give raw data or particular details of the experimental operation as long as the request is legitimate.
