# Supplementary material for: Cepharanthine, a regulator of keap1-Nrf2, inhibits gastric cancer growth through oxidative stress and energy metabolism pathway
Source: Cell Death Discov. 2023 Dec 12;9:450. doi: 10.1038/s41420-023-01752-z (PMC10716385; doi:10.1038/s41420-023-01752-z)
Supplement: Supplementary file 1 — Supplementary Material [file 41420_2023_1752_MOESM1_ESM.docx]

**FIGURE LEGENDS**

**Figure1S**

Fig. 1S. (A) MFC cell line was treated with CEP for 48 h. Morphology was observed by live cell photography—scale bar: 100 μm. (B) MFC cell line was treated with different concentrations of CEP for 48 h. The cell viability was detected by MTT assay (n = 3). (C) Cloning of MFC cell line treated with CEP (1.25, 2.5, and 5 μmol/L). Quantify the clonal formation of MFC cell by measuring the absorbance of the solution obtained by dissolving crystal violet in glacial acetic acid (n = 3). (D) A scratch assay was performed to determine the migration of MFC cell treated with CEP (1.25, 2.5, and 5 μmol/L) for 48 h. Quantification of scratch images by calculating the area of cell migration (n = 3). Data were expressed as mean ± SD, *p < 0.05, **p < 0.01, ***p < 0.001, and ****p < 0.0001, with significant differences from the control group.

**Figure2S**

Fig. 2S. (A) The cell cycle of MFC cell line treated with CEP (1.25, 2.5, and 5 μmol/L) for 48 h was analyzed by flow cytometry. The percentage of MFC cell in different phases (n = 3). (B) After CEP treatment, a western blot was used to detect CyclinD1 and CDK2 levels in MFC cell. Protein levels were standardized using GAPDH levels. (C) Cell apoptosis detected by Hoechst 33342 staining. MFC cell was exposed to CEP (1.25, 2.5, and 5 μmol/L) for 48 h, and fluorescence images were taken. Scale bar: 100 µm. (D) Flow cytometry assessed cell apoptosis in MFC cell treated with CEP (1.25, 2.5, and 5 μmol/L) for 48 h. (E) Western blot was used to determine the levels of apoptosis-related proteins in MFC cell after CEP treatment. CEP treatment significantly increased the ratios of Cleaved caspase-3/capase-3, Cleaved caspase-9/caspase-9, significantly increased Bax, Bad protein levels, and significantly down-regulated Bcl-2 and PARP1 (n = 3). The protein levels were standardized using GAPDH levels. Data were expressed as mean ± SD, *p < 0.05, **p < 0.01, ***p < 0.001, and ****p < 0.0001, with significant differences from the control group.

**Figure3S**

Fig. 3S. (A) Intracellular ROS was detected by DCFH-DA staining (green) and DHE staining (red). MFC cell was treated with CEP (1.25, 2.5, and 5 μmol/L) for 48 h, and fluorescence images were captured (n = 3). Scale bar: 100 μm. (B) The mitochondrial membrane potential was measured with JC-1 dye. MFC cell was treated with CEP for 48 h—scale bar: 100 μm. (C-D) MFC cell was treated with 1.25, 2.5, and 5 μmol/L CEP for 48 h. The protein expression of Nrf2, Keap1, GCLM, NQO1, HMOX1, cyto-Nrf2 and nucl-Nrf2 was measured by western blot (n = 3). (E) MFC cell was treated with 2.5 μmol/L CEP for 48 h. Measurement of Nrf2 protein nuclear translocation by laser confocal microscopy. Scale bar: 50 μm. The data were expressed as means ± SD; *p < 0.05, **p < 0.01, ***p < 0.001, and****p < 0.0001, significantly different from the control group.

**Supplementary Figure. S1**


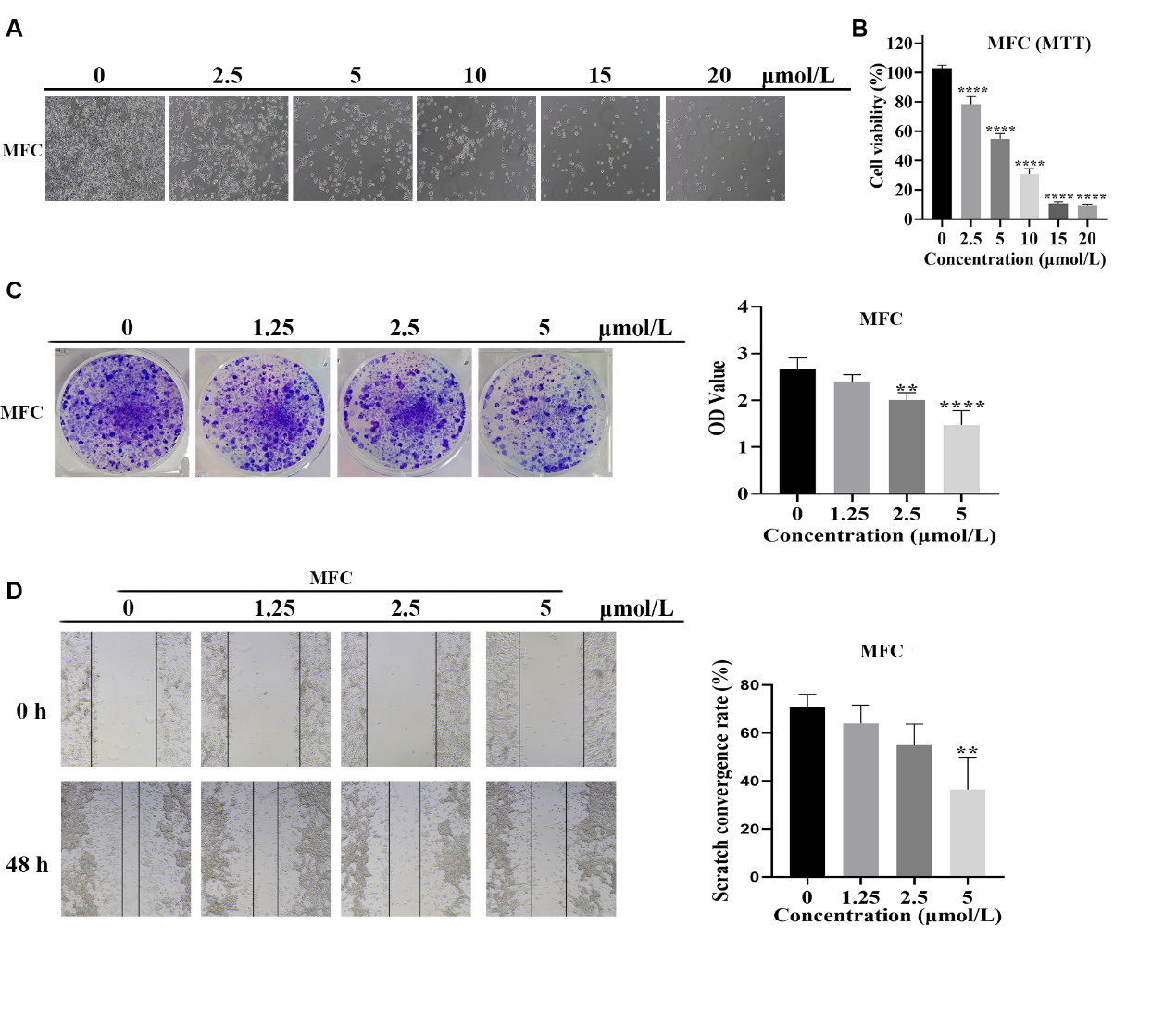


**Supplementary Figure. S2**


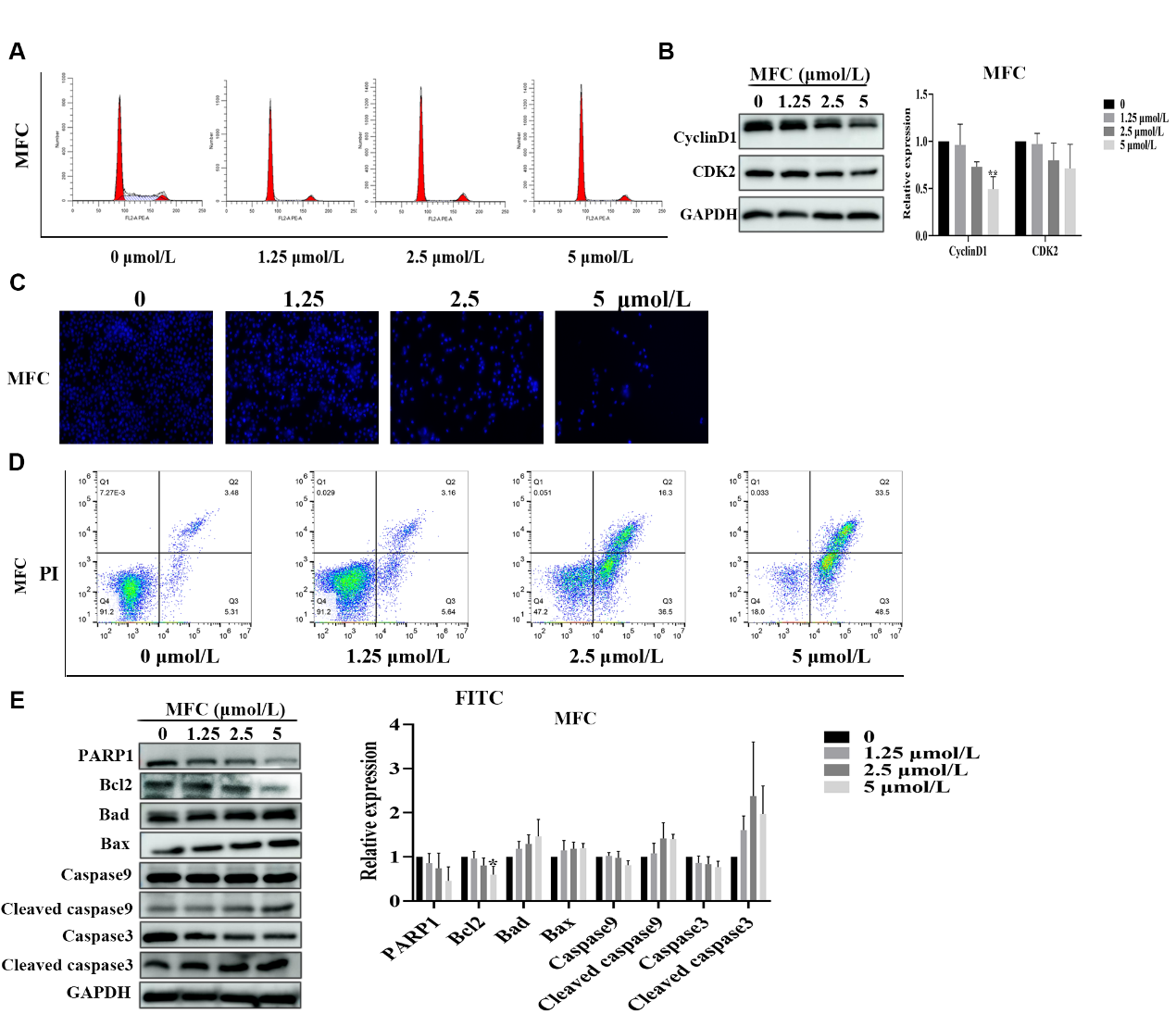


**Supplementary Figure. S3**

**
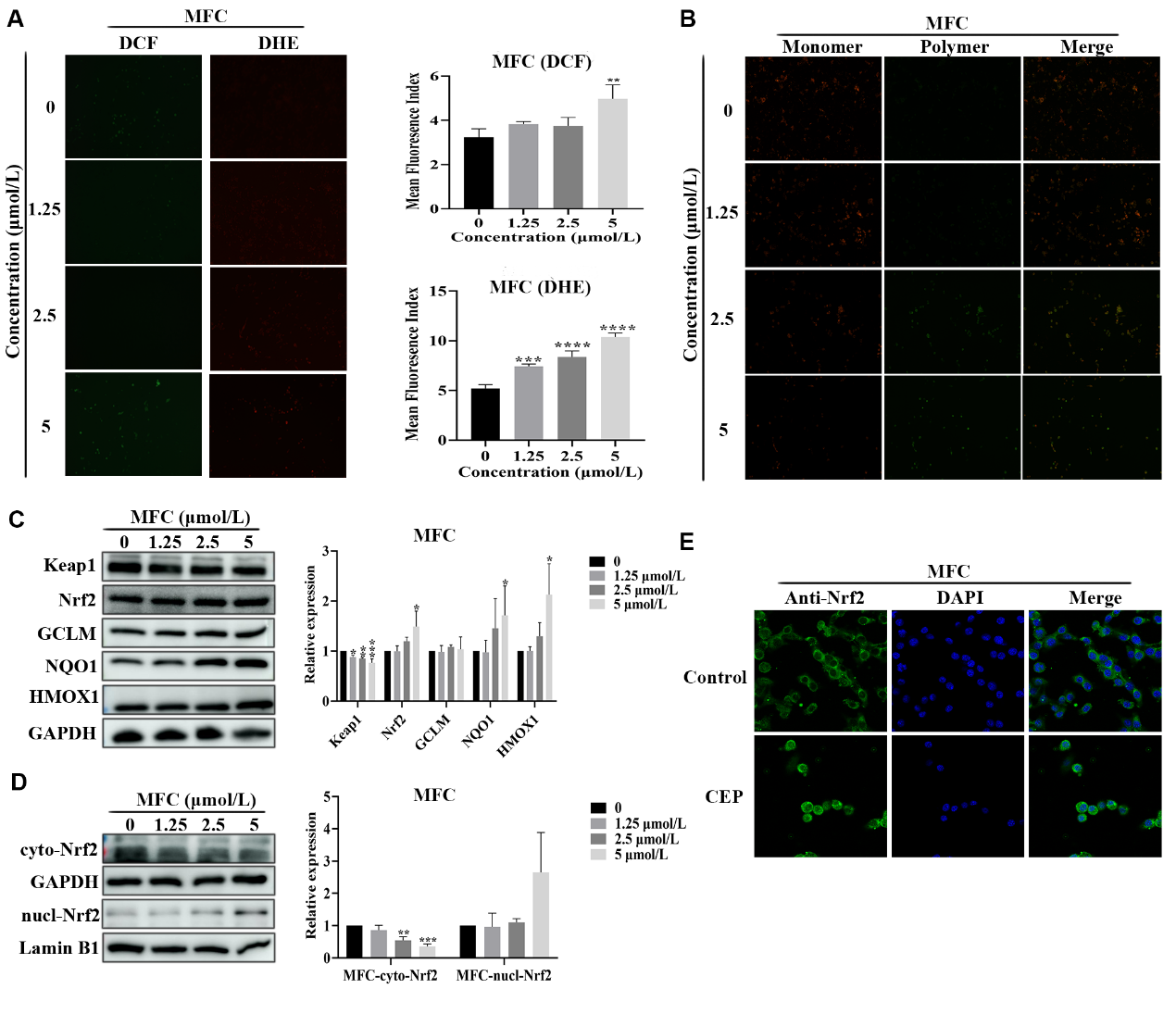
**
